# Effects of High-Frequency Oscillatory Ventilation With Volume Guarantee During Surfactant Treatment in Extremely Low Gestational Age Newborns With Respiratory Distress Syndrome: An Observational Study

**DOI:** 10.3389/fped.2021.804807

**Published:** 2022-03-03

**Authors:** Milena Tana, Angela Paladini, Chiara Tirone, Claudia Aurilia, Alessandra Lio, Anthea Bottoni, Simonetta Costa, Eloisa Tiberi, Roberta Pastorino, Giovanni Vento

**Affiliations:** ^1^Unità Operativa Complessa di Neonatologia, Fondazione Policlinico Universitario A. Gemelli IRCCS, Rome, Italy; ^2^Section of Hygiene, Institute of Public Health, Università Cattolica del Sacro Cuore, Rome, Italy; ^3^Department of Woman and Child Health and Public Health, Università Cattolica del Sacro Cuore, Rome, Italy

**Keywords:** HFOV, volume guarantee, ELGAN, respiratory distress syndrome, lung recruitment

## Abstract

**Objective:**

To evaluate the effect of volume guarantee (VG) combined with high-frequency oscillatory ventilation (HFOV) on respiratory and other physiological parameters immediately after lung recruitment and surfactant administration in HFOV elective ventilated extremely low gestational age newborns (ELGAN) with respiratory distress syndrome (RDS).

**Design:**

Observational study.

**Setting:**

Tertiary neonatal intensive care unit.

**Patients:**

Twenty-two ELGANs of 25.5 ± 1.1 weeks of gestational age requiring invasive mechanical ventilation and surfactant administration for RDS during the first 6 h of life.

**Interventions:**

All infants intubated in delivery room, were managed with elective HFOV and received surfactant after a lung recruitment manoeuver. Eleven infants received HFOV + VG and were compared with a control group of 11 infants receiving HFOV alone. HFOV was delivered in both groups by Dräger Babylog VN500 ventilator (Dräger, Lubeck, Germany).

**Main Outcome Measures:**

Variations and fluctuations of delivered high-frequency tidal volume (VT_hf_), fluctuation of pressure amplitude (ΔP) and partial pressure of CO_2_ (pCO_2_) levels after recruitment manoeuver and immediately after surfactant administration, in HFOV + VG vs. HFOV ventilated infants.

**Results:**

There were no significant differences in the two groups at starting ventilation with or without VG. The mean applied VT_hf_ per kg was 1.7 ± 0.3 ml/kg in the HFOV group and 1.7 ± 0.1 ml/kg in the HFOV + VG group. Thirty minutes after surfactant administration, HFOV group had a significant higher VT_hf_/Kg than HFOV + VG (2.1 ± 0.3 vs. 1.6 ± 0.1 ml/kg, *p* < 0.0001) with significantly lower pCO_2_ levels (43.1 ± 3.8 vs. 46.8 ± 1.5 mmHg, *p* = 0.01), 54.4% of patients having pCO_2_ below 45 mmHg. Measured post-surfactant ΔP values were higher in HFOV group (17 ± 3 cmH_2_O) than in HFOV + VG group (13 ± 3 cmH_2_O, *p* = 0.01).

**Conclusion:**

HFOV + VG maintains pCO_2_ levels within target range and reduces VT_hf_ delivered variations more consistently than HFOV alone after surfactant administration.

## Introduction

Despite a shift toward non-invasive respiratory support, mechanical ventilation and surfactant administration in the first hours of life may be life-saving in preterm infants with respiratory distress syndrome (RDS), especially in extremely low gestational age newborns (ELGAN) (gestational age ≤ 27 weeks) (1, 2). In the last years, high-frequency oscillatory ventilation (HFOV) has been increasingly used in preterm infants with RDS, because early HFOV could reduce risk of bronchopulmonary dysplasia (BPD) (3), especially if associated with an open lung strategy (4, 5).

HFOV offers highly effective oxygenation and clearance of waste gas, despite use of tidal volumes at or below dead space volume (6, 7). The CO_2_ diffusion (gas transport) coefficient (DCO_2_) is a vital variable in HFOV and is calculated as frequency times the square of tidal volume during HFOV (VT_hf_) (8, 9). Recently, weight-corrected DCO_2_ ([ml/kg]^2^/s) has been proposed to reduce inter-individual variability (10). Consequently, VT_hf_ is crucial for CO_2_ elimination with a larger impact in comparison to tidal volume during conventional mechanical ventilation. The VT_hf_ is determined by the amplitude of the pressure oscillations (ΔP), and it is delivered to the lungs around a constant mean airway pressure (MAP). As the same ΔP can be associated with very different level of chest oscillations and VT_hf_ in different babies, and even in the same baby over the course of pulmonary disease, the same ΔP can result in very large variations of VT_hf_ and unexpected variations of CO_2_ removal (11, 12).

Volume guarantee (VG), a form of volume-targeted ventilation, is known to improve neonatal prognosis and has been a well-established respiratory management for preterm infants when combined with conventional ventilation (13–16). In the last few years, volume-guarantee modality has been combined with HFOV (HFOV+VG), demonstrating an attenuation of the fluctuations in SpO_2_ and CO_2_, which can prevent hypoxemia and hypocapnia (17). Iscan et al. compared HFOV and HFOV + VG in the same preterm infants (24–32 weeks' gestation) with RDS, intubated within the first 6 h of life and treated with surfactant. The HFOV + VG period reported a reduction in fluctuations of VT_hf_, in the number of out-of-target pCO_2_ levels and hypoxia events compared with HFOV group (18). This is confirmed in detail by Belteki et al. (19) recording every VT_hf_ from the ventilator and showing that the tidal volume of the oscillations varies in the short term but is kept very close to the long term objective. However, according to the present literature, a specific optimal VT_hf_ has not yet been recommended, as it varies with the instantaneous characteristics of individuals and frequencies (20, 21).

In our previous study aimed to evaluate the changes in end-expiratory lung volume during an oxygenation-guided stepwise recruitment procedure in HFOV without VG, mean VThf significantly increased between the start and the end of the recruitment with a slight not significant reduction of mean pCO_2_ values, possible effect of the increased lung compliance (22).

To our knowledge, changes in VThf and the effects of HFOV + VG during surfactant administration in preterm infants with RDS have not been thoroughly studied.

HFOV open lung recruitment maneuver and surfactant administration are followed by improved alveolar ventilation potentially resulting in volutrauma and hypocapnia (23–25). Volutrauma can contribute to BPD, and hypocapnia can alter cerebral blood flow increasing the risk of intra-ventricular hemorrhage (IVH), periventricular leukomalacia (PVL), and poor neurodevelopmental outcomes (24–30). In newborn animal model of surfactant deficiency, the use of HFOV combined with VG ventilation demonstrated benefits of setting the VT_hf_ instead of ΔP to modify CO_2_ removal from the lung (31). Volume guarantee combined with HFOV may be clinically advantageous, especially in conditions in which lung compliance can change rapidly, such as during and immediately after surfactant treatment.

The aim of the present study was to investigate the effect of VG combined to HFOV on respiratory and other physiological parameters in HFOV electively ventilated ELGAN with RDS immediately after surfactant administration, when lung compliance can change rapidly.

## Materials and Methods

### Patients

This is a single center, observational study conducted in our third level neonatal intensive care unit (NICU). This study was approved by the Ethics Committee of Fondazione Policlinico Universitario A. Gemelli IRCCS, Roma—Università Cattolica del Sacro Cuore (ID 4425). The inclusion criteria were as follows: inborn premature infants with a gestational age (GA) between 24 and 27 weeks (ELGAN), requiring endotracheal intubation at birth with a diagnosis of RDS, electively HFOV ventilated and receiving surfactant treatment in the first 6 h of life, and after a recruitment maneuver as open lung strategy using oxygenation as indirect marker for lung volume (5). In our NICU, we started using HFOV + VG during clinical care in June 2017, because of technical improvement led by Dräger specialists and the increased experience and skills of the entire neonatal staff with the Dräger Babylog VN500 ventilator (Dräger Medical, Lubeck, Germany), using the HFOV + VG modality.

In fact, before June 2017, all ELGANs that needed mechanical ventilation were managed by HFOV without VG. A historical control group of ELGANs, born between June 2016 and June 2017 and electively ventilated in HFOV, was then selected and compared with a group of ELGANs prospectively studied in the period June 2017–June 2018, electively ventilated in HFOV + VG.

**Figure 1:**
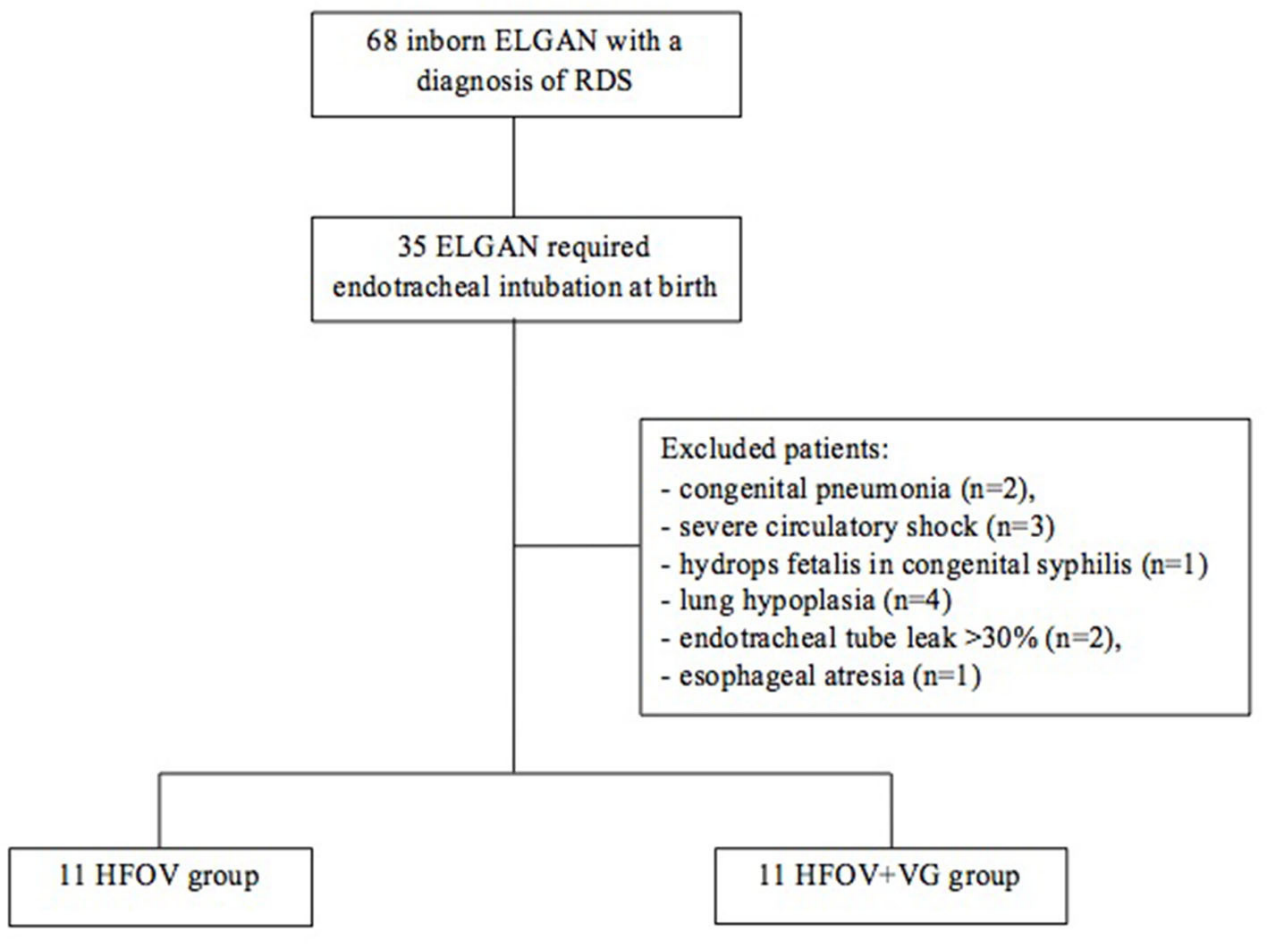
Flow diagram.

Exclusion criteria were as follows: outborn patients, presence of major congenital malformations, hydrops fetalis inherited disorders of metabolism, congenital pneumonia (positive bronchoalveolar lavage fluid culture at birth), pulmonary hypertension (confirmed by echocardiography), suspected pulmonary hypoplasia (based on clinician interpretation of a chest radiograph with small and hypoplastic-appearing lungs; premature rupture of membranes and/or oligohydramnios documented on antenatal ultrasound 3 or more weeks prior to delivery), severe circulatory shock (prolonged capillary filling, reduced strength of peripheral pulses, cool skin, lethargy, hypotension, oliguria, increasing lactate concentrations, and metabolic acidosis), and >20% endotracheal tube leak to negate the effect of leakage (Figure 1).

### HFOV and Open Lung Ventilation Strategy

HFOV is the primary mode of mechanical ventilation in our NICU to manage preterm newborns with GA ≤ 27 weeks and/or BW < 1,000 g affected by RDS because of the results of our previous randomized controlled trial comparing the effects of HFOV vs. conventional mechanical ventilation (32).

In all studied infants, HFOV was delivered by Dräger Babylog VN500 (Dräger Medical, Lubeck, Germany) and started at a MAP of 8–10 cmH_2_0, a frequency of 15 Hz, and an inspiratory/espiratory ratio of 1:2 (I:E = 1:2). The FiO_2_ was initially set to ensure adequate oxygen saturation (SpO_2_ 89–94%).

In all infants, frequency (15 Hz) and I:E = 1:2 remained unchanged during the studied ventilation periods.

The goals of respiratory management were to maintain pH 7.30–7.45, pCO_2_ 45–55 mmHg (5.9–7.2 kPa), pO_2_ 50–70 mm Hg (6.6–9.3 kPa), and SpO_2_ 89–94%.

In all studied infants, HFOV or HFOV + VG was combined with an open lung ventilation strategy aiming to recruit and stabilize most collapsed alveoli/sacculi, using oxygenation as an indirect parameter for lung volume (33, 34).

The MAP was increased stepwise by 1–2 cmH_2_O every 2–3 min as long as SpO_2_ improves. The FiO_2_ was reduced stepwise, keeping SpO_2_ within the target range. The recruitment procedure was stopped if the FiO_2_ did not exceed 0.25 or if oxygenation no longer improved or there were signs of lung hyperinflation (capillary refill time >3 s and/or hypotension). The corresponding MAP was the pre-surfactant opening continuous distending pressure (CDP_O_). Next, the MAP was reduced stepwise by 1–2 cmH_2_O every 2–3 min until the SpO_2_ was deteriorated (of at least 2–3 points), and the corresponding MAP was the pre-surfactant closing pressure (CDP_C_). Finally, the lung was recruited again by returning to the known CDP_O_ for 2–3 min and then stabilized setting MAP to 2 cmH_2_O above the CDP_C_ at pre-surfactant optimal pressure (CDP_OPT_) for at least 3 min.

Surfactant was then administered at the CDP_OPT_ (Curosurf, Chiesi) *via* a closed system catheter at a dose of 200 mg/kg.

Surfactant treatment in open lung HFOV ventilated preterm infants with RDS causes rapid increase (minutes) and subsequent stabilization of lung volume and increases maximal compliance of the lung, but at lower airway pressures (23).

Consequently, to avoid possible overdistension and to profit from the increased compliance after surfactant treatment, after a stabilization period of 5–10 min, MAP was reduced by 1–2 cmH_2_O and CDP_C_, CDP_O_, and CDP_OPT_ were once more determined post-surfactant.

### HFOV and HFOV + VG Ventilation Strategy

In the HFOV group, ΔP was initially set at 15 cm H_2_O and then increased if necessary until the infant's chest was seen to be visibly vibrating. Operators adjusted ΔP up or down in increments of 1–2 cm H_2_O as necessary, if pCO_2_ values were outside the target range, to achieve a VT_hf_ of 1.5–2.0 ml/kg.

In the HFOV + VG group, the target range of VT_hf_ was 1.5–1.8 ml/kg and ΔP_max_ limit was set 15–20% above the average ΔP needed to achieve it, frequency was set at 15 Hz, and inspiratory/espiratory ratio was 1:2. The set VT_hf_/Kg was adjusted by the clinicians up or down in increments of 0.2 ml/kg as frequently as necessary, if pCO_2_ values were outside of the target range.

All patients received a loading dose of caffeine (20 mg/kg) immediately after admission to the NICU, then maintenance therapy (35), and Remifentanil by continuous intravenous infusion at a dose of 0.075 μg/kg/min to provide analgesia during HFOV while preserving spontaneous respiratory activity (36).

### Data Collection

Data of the historical HFOV group were collected from ventilation sheets where doctors of our Unit usually report vital signs and respiratory and ventilation parameters during surfactant administration in ventilated patients.

Demographic data on patient and maternal characteristics were collected from each patient.

Information was collected on MAP, FiO_2_, ΔP, VT_hf_ per weight, frequency, DCO_2_ per weight, SpO_2_, and pCO_2_ at different time points: start of ventilation in the NICU, at the pre-surfactant time (after the stabilization period at CDP_OPT_ pre-surfactant time), and at 30 min post-surfactant (after the stabilization period at CDP_OPT_ post-surfactant time). To assess the severity of lung disease at the start of ventilation, the oxygenation index (OI): (MAPxFiO_2_/PaO_2_x100) was calculated for each patient.

In all patients, a capillary blood sample after adequate heel warming was obtained for gas analysis at each of the three study phases (baseline, pre-surfactant, and post-surfactant).

### Statistical Analysis

Values were expressed as mean and SD or median and range for continuous variables or absolute frequency and percentages for categorical variables. Continuous variables were compared with parametric (Student's *t*-test) or non-parametric (Mann–Whitney *U*-test) tests, as appropriate. Categorical variables were compared by using a two-tailed Fisher's exact test. A 2-tailed value of *p* < 0.05 was considered significant. Pearson's correlation analysis was performed to determine the correlations between selected parameters.

Data were analyzed using commercial statistical software (GraphPad Prism version 8.0.0, San Diego, California, USA).

## Results

Between 1 June 2016 and 30 June 2018, a total of 68 inborn ELGAN with a diagnosis of RDS requiring on-going intensive care were admitted to our NICU. Thirty-five of these ELGAN required endotracheal intubation at birth and were electively ventilated by HFOV.

Thirteen of these infants were excluded for the following reasons: congenital pneumonia (*n* = 2), severe circulatory shock (*n* = 3, two septic shocks, one recipient baby of twin-to-twin transfusion syndrome), hydrops fetalis in congenital syphilis (*n* = 1), severe pulmonary hypertension in lung hypoplasia secondary to prolonged premature rupture of membranes (*n* = 4), >30% endotracheal tube leak (*n* = 2), and esophageal atresia (*n* = 1).

Of the remaining 22 patients with 25.5 ± 1.1 weeks of GA and 721 ± 115 g of birth weight, 11 were born between 1 June 2016 and 15 June 2017 and electively ventilated in HFOV (HFOV group), and 11 were born between 15 June 2017 and 30 June 2018 and electively ventilated in HFOV combined with VG (HFOV + VG group). No significant differences were observed between the two groups in terms of demographic and clinical characteristics (Table 1), including the severity of the lung disease, as demonstrated by oxygenation index (OI) (MAPxFiO_2_/PaO_2_x100 values at baseline; Table 2). All patients were intubated with 2.5 mm endotracheal tube. In all infants, frequency (15 Hz) and inspiratory/espiratory ratio of 1:2 remained unchanged during the studied ventilation periods.

**Table 1:** Patient and maternal demographics and neonatal characteristics at birth.

	**HFOV (*n =* 11)**	**HFOV+VG (*n =* 11)**	***p***
Gestational age, weeks	25.8 ± 1.0	25.2 ± 1.2	0.20
Birth weight, g	754 ± 74	688 ± 141	0.20
Complete course of antenatal steroids^a^	5 (45)	8 (44)	1
5-min Apgar score	7 [7–9]	7 [4–9]	0.50
SGA	2 (18)	2 (18)	1
Male	5 (45)	6 (54)	1
Premature rupture of membranes >12 h	4 (36)	5 (45)	1
Delivery by cesarean section	9 (82)	10 (91)	1
Surfactant, hours of life	2.6 ± 1.7	2.7 ± 1.8	0.86

*Values expressed as mean ± SD, median [range] and number (percent)*.

a *A complete course of antenatal steroids was defined as two doses of betamethasone administered more than 24 h but no more than 7 days before delivery. SGA, small for gestational age*.

**Table 2:** Ventilator settings, ventilation at baseline, and pre-surfactant and post surfactant time.

	**HFOV (*n* = 11)**	**HFOV+VG (*n* = 11)**	***p***
**Baseline**			
MAP (cmH_2_O)	9.7 ± 0.5	9.5 ± 0.8	0.35
FiO_2_	0.43 ± 0.10	0.46 ± 0.10	0.96
ΔP (cmH_2_O)	20 ± 3	19 ± 1	0.32
VT_hf_ (ml/kg)	1.7 ± 0.3	1.7 ± 0.1	0.53
DCO_2_ (ml^2^/kg^2^/s)	46.5 ± 18.0	42.2 ± 16.1	0.46
pCO_2_ (mmHg)	50.5 ± 4.0	50.0 ± 3.2	0.73
OI	8.0 ± 2.3	8.4 ± 2.0	0.35
**Pre-surfactant**			
MAP (cmH_2_O)	13.7 ± 1.4	13.9 ± 1.4	0.77
FiO_2_	0.25 ± 0.01	0.25 ± 0.01	1
ΔP (cmH_2_O)	20 ± 3	18 ± 2	0.14
VT_hf_ (ml/kg)	1.8 ± 0.3	1.6 ± 0.1	**0.03**
DCO_2_ (ml^2^/kg^2^/s)	51.6 ± 16.3	39.4 ± 5.1	**0.03**
pCO_2_ (mmHg)	48.4 ± 3.2	48.0 ± 2.2	0.76
**Post-surfactant**			
MAP (cmH_2_O)	8.7 ± 0.7	8.6 ± 0.7	0.88
FiO_2_	0.21 ± 0.01	0.21 ± 0.01	0.75
ΔP (cmH_2_O)	17 ± 3	13 ± 3	**0.005**
VT_hf_ (ml/kg)	2.1 ± 0.3	1.6 ± 0.1	**<0.0001**
DCO_2_ (ml^2^/kg^2^/s)	69.5 ± 16.4	39.5 ± 5.8	**<0.0001**
pCO_2_ (mmHg)	43.1 ± 3.8	46.8 ± 1.5	**0.006**

*Values expressed as mean ± SD. MAP, mean airways pressure; VT_hf_ (ml/kg), tidal volume HFOV; DCO_2_, coefficient of gas transport; FiO_2_, fraction of inspired oxygen; OI, oxygenation index (MAP x FiO_2_/PaO_2_x 100)*.

*Bold values are the statistically significant p-values*.

The study population ventilator parameters at baseline are shown in Table 2. There were not significant differences in the 2 groups at baseline between ventilation modes in terms of MAP, ΔP, VT_hf_ per kg, DCO_2_, pCO_2_, frequency, and I:E. The mean applied VT_hf_/Kg was 1.7 ± 0.3 ml/kg in the HFOV group and 1.7 ± 0.1 in the HFOV + VG group.

**Figure 2:**
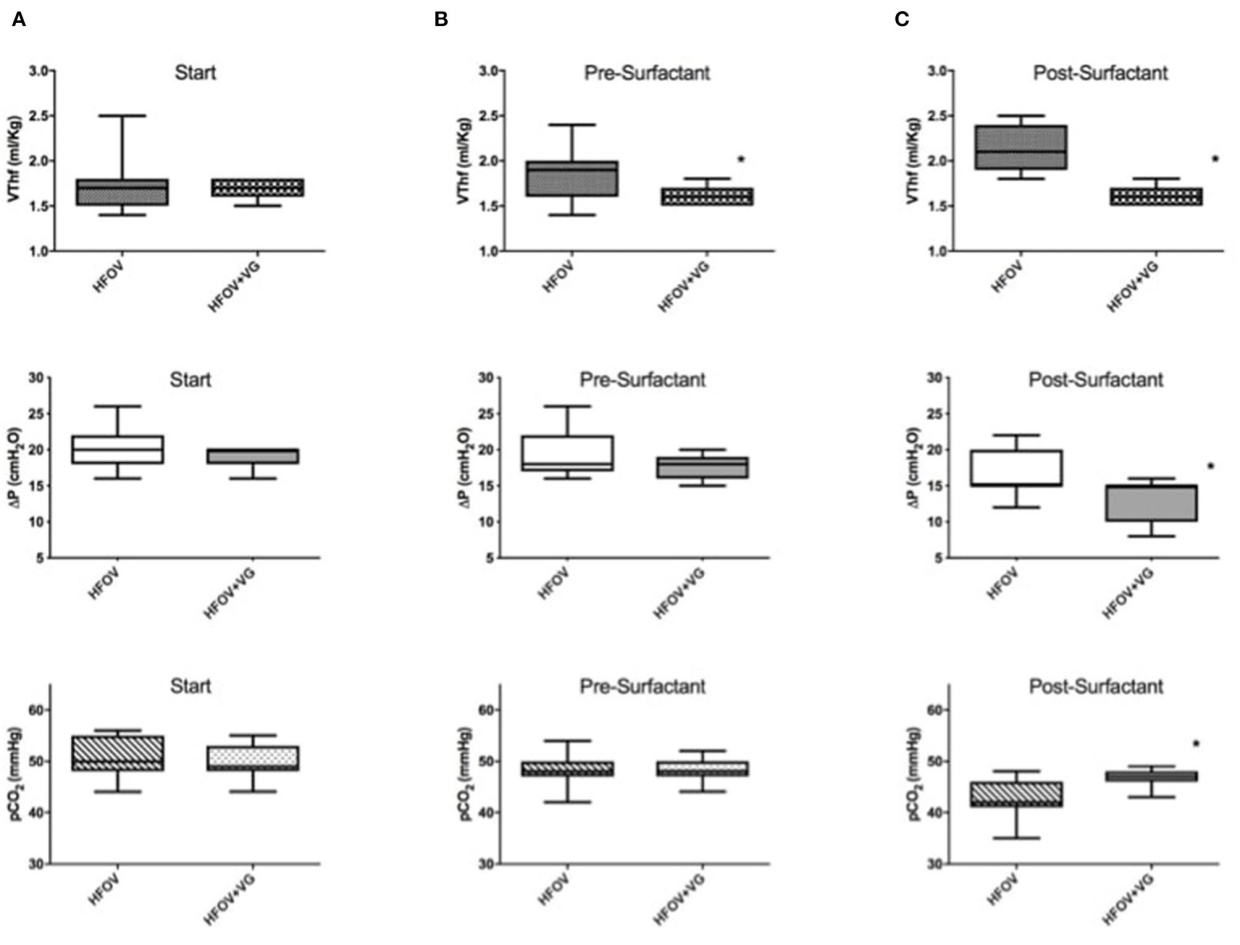
Evaluation of VT_hf_ (ml/kg), amplitude level (ΔP) and pCO_2_ in HFOV and HFOV + VG groups. **(A)** At start: no significant differences of the three parameters between the two groups were observed. **(B)** Pre-surfactant administration (after completing the lung recruitment maneuver): there is a significant difference between the two groups in terms of VT_hf_/kg (*p* = 0.03). **(C)** Post-surfactant administration: there are significant differences between the two groups in terms of VT_hf_/kg (*p* < 0.0001), ΔP (*p* = 0.005), and pCO_2_ (*p* = 0.006). ^*^Represents statistically significant differences.

Considering data after the recruitment maneuver (i.e., pre-surfactant administration), HFOV group had a significantly higher VT_hf_/Kg than HFOV + VG (1.8 ± 0.3 vs. 1.6 ± 0.1, *p* = 0.03) with higher VT_hf_/Kg variability (in terms of SD), not corresponding to a significant difference in pCO_2_ values (Figure 2 and Table 2).

After surfactant administration, HFOV group, compared to HFOV + VG group, had significantly higher VT_hf_/Kg (2.1 ± 0.3 vs. 1.6 ± 0.1; *p* < 0.0001) with significantly lower pCO_2_ values (43.1 ± 3.8 vs. 46.8 ± 1.5; *p* = 0.006). Moreover, six patients of HFOV group (54.4%) reached a pCO_2_ value below target of 45 mmHg and one patient with pCO_2_ below 35 mmHg of pCO_2_ (Figure 2).

**Figure 3:**
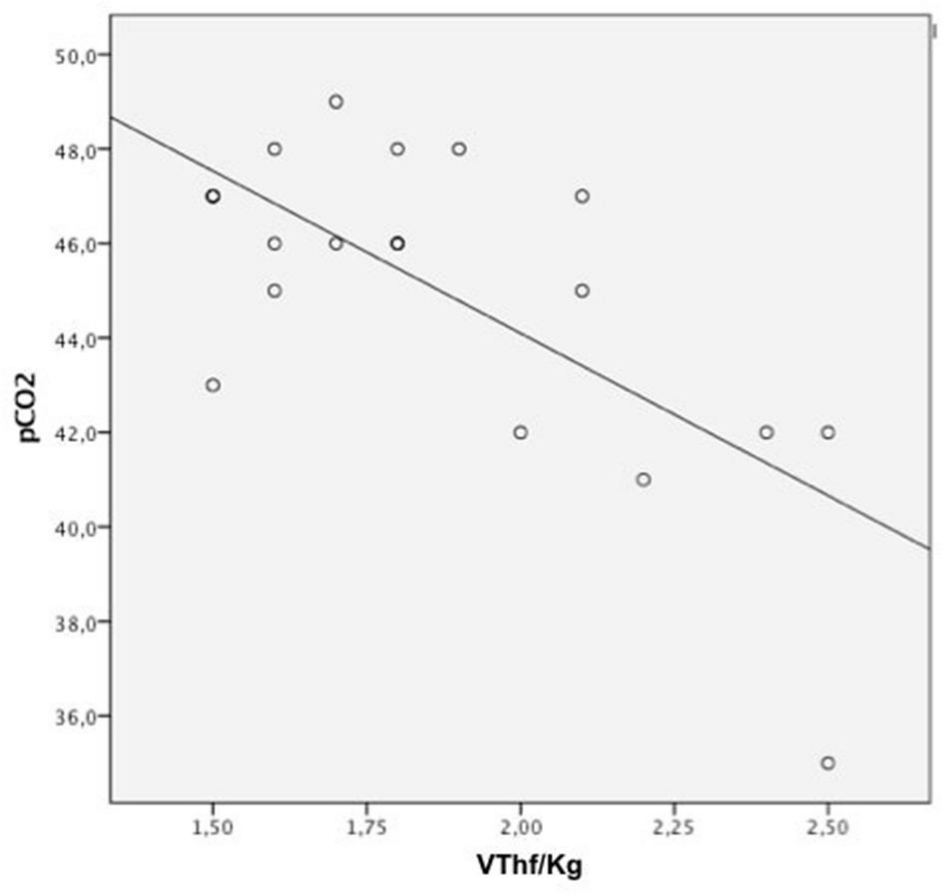
Pearson correlation analysis between VThf/kg, pCO2 significant correlation between all VThf/kg and pCO2 levels (Pearson coefficient: *r* = −0.69, *p* < 0.0001).

A significant negative correlation was found between VT_hf_/Kg values and the corresponding pCO_2_ after surfactant administration in all patients of both groups (*r*: −0.69; *n*:21, *p* < 0.0001; Figure 3).

In both groups, the ΔP significantly decreases after surfactant administration, but comparing the 2 groups, the ΔP post-surfactant is lower in HFOV + VG with 13 ± 3 cm H_2_O vs. 17 ± 3 cm H_2_O; p = 0.01 (Figure 2 and Table 2).

Data on weight corrected gas transport coefficient (DCO_2_) in the HFOV and HFOV + VG groups show a mean DCO_2_ measurement significantly different, with higher values in HFOV both pre-surfactant (51.6 ± 16.3 vs. 39.4 ± 5.1; *p* = 0.03) and post-surfactant (69.5 ± 16.4 vs. 39.5 ± 5.8; *p* < 0.0001).

## Discussion

To our knowledge, this is the first paper of comparison between HFOV and HFOV + VG during surfactant administration in extremely preterm neonates with RDS electively HFOV ventilated. Considering the last literature, a multicentre, randomized, controlled trial, IN-REC-SUR-E (37), demonstrated how surfactant administration after lung recruitment with HFOV decreased the need for MV in the first 72 h of life in extremely preterm infants compared to standard IN-SUR-E technique. Volume recruitment maneuver improves surfactant distribution and pulmonary gas exchange as also seen in animal studies (38). Conditions where alveolar ventilation is improved and lung compliance changes rapidly, like during open lung HFOV surfactant treatment, can result in volutrauma and hypocapnia (23–25). During HFOV, the same pressure amplitude and frequency can result in different chest oscillations and tidal volume due to changes in lung mechanics and patient–ventilator interactions. Data published on HFOV + VG in preterm infants constantly report the feasible use and a better maintenance of VT_hf_ and pCO_2_ in the target range (19–22). The aim of our study was to evaluate VG combined to HFOV during surfactant administration.

Avoiding volutrauma is a desirable goal in ventilation, with immediate and long term benefits associated to the prevention of both lung injury with BPD and hypocapnia that can alter cerebral blood flow with increased risk of intra-ventricular hemorrhage (IVH), periventricular leukomalacia (PVL), and poor neurodevelopmental outcomes (26–30).

In our study variations of VT_hf_ and ΔP were evaluated during surfactant administration, i.e., a rapidly lung compliance change phase, and the low and stable VT_hf_/Kg observed in HFOV + VG patients cause a significant low DCO_2_ values in this group. The mean VT_hf_/Kg levels used in our study, corresponding to normocapnic blood gases, were 1.5–1.8 ml/kg. We used a constant frequency of 15 Hz to reduce the determinants of DCO_2_. As Mukerji et al. demonstrated in artificial lung model, frequency has a direct relationship with CO_2_ elimination when tidal volume is fixed. Using low delivered tidal volumes and high frequencies may allow for improved ventilation efficacy, while minimizing lung injury (39).

Recently, it is reported that it is possible to use lower delivered tidal volumes during HFOV combined with VG and higher frequencies to allow minimizing lung injury. In experimental models, the protective effect of HFOV + VG has been proved when using smaller volumes and very high frequencies (40) which have been successfully used in preterm newborns in a pilot study of González-Pacheco (41). In a recent retrospective cohort study investigating the high-frequency parameters corresponding with adequate ventilation, the median high-frequency tidal volume corrected by birth weight was 1.63 ml/kg for frequencies 15 Hz, and tidal volumes were inversely correlated with frequencies used (21).

To date, there are no starting ventilation parameters defined for VG and frequency in HFOV ventilation. Few studies support the need for higher VT_hf_ during HFOV ranging between 1.75 and 1.90 ml/kg using a constant frequency of 10 Hz (18, 42). Another study in an almost homogeneous group of preterm infants, already treated with surfactant, used an average VT_hf_ of 1.64 ± 0.25 ml/kg (20).

In our highly homogeneous population of extremely preterm infants with RDS evaluated before and after surfactant administration, an HFOV + VG starting ventilator setting with VT_hf_ 1.5–1.8 ml/kg and a respiratory rate of 15 Hz has been demonstrated to be safe and efficacious.

This is a retrospective study, so the method of data recording is a first limitation. Another important limitation is the small number of patients evaluated in two different study periods. However, in our unit, HFOV + VG is nowadays the elective and routine modality of invasive ventilation and it is not possible to perform a randomized controlled trial. Finally, our patients received surfactant at a mean age of 2.5 h. The duration of the HFOV recruitment maneuver (average 20–30 min) may have contributed to the delay in surfactant administration, which is usually recommended soon after intubation.

In conclusion, although the optimal initial value of VThf cannot be exactly known because it can be influenced by prenatal conditions, sedation, and spontaneous breathing, in a selected population we can suggest safety values of VT_hf_/kg and frequency to obtain normocapnia.

Our results show that HFOV + VG, compared to HFOV alone, avoids increasing VT_hf_ just after surfactant administration, reduces VT_hf_ fluctuations (as demonstrated by lower SD of mean VT_hf_ values), and reduces large pCO_2_ excursions and risk of hypocapnia.

Due to the lower ΔP and VThf values and to the reduced fluctuation of pCO_2_ levels, HFOV combined with VG appears to be suitable for extremely preterm infants, compared to HFOV alone, in the management of acute neonatal RDS before and after surfactant administration.

## Data Availability Statement

The original contributions presented in the study are included in the article/supplementary material, further inquiries can be directed to the corresponding author/s.

## Ethics Statement

The studies involving human participants were reviewed and approved by Comitato Etico, Policlinico Universitario, Agostino Gemelli—Roma. Written informed consent to participate in this study was provided by the participants' legal guardian/next of kin.

## Author Contributions

MT, AP, and GV conceptualized and edited the final manuscript. RP supervised the statistical analysis. MT, AP, CT, CA, AL, AB, SC, and ET collaborated in clinical practice and data collection. All authors contributed to the article and approved the submitted version.

## Conflict of Interest

The authors declare that the research was conducted in the absence of any commercial or financial relationships that could be construed as a potential conflict of interest.

## Publisher's Note

All claims expressed in this article are solely those of the authors and do not necessarily represent those of their affiliated organizations, or those of the publisher, the editors and the reviewers. Any product that may be evaluated in this article, or claim that may be made by its manufacturer, is not guaranteed or endorsed by the publisher.
